# The Role of the Glycocalyx in the Pathophysiology of Subarachnoid Hemorrhage-Induced Delayed Cerebral Ischemia

**DOI:** 10.3389/fcell.2021.731641

**Published:** 2021-09-03

**Authors:** Hanna Schenck, Eliisa Netti, Onno Teernstra, Inger De Ridder, Jim Dings, Mika Niemelä, Yasin Temel, Govert Hoogland, Roel Haeren

**Affiliations:** ^1^Department of Neurosurgery, Maastricht University Medical Center, Maastricht, Netherlands; ^2^Department of Neurosurgery, Helsinki University Hospital, Helsinki, Finland; ^3^Department of Neurology, Cardiovascular Research Institute Maastricht, Maastricht University Medical Center, Maastricht, Netherlands

**Keywords:** subarachnoid hemorrhage, aneurysm, delayed cerebral ischemia, glycocalyx, pathophysiology, review

## Abstract

The glycocalyx is an important constituent of blood vessels located between the bloodstream and the endothelium. It plays a pivotal role in intercellular interactions in neuroinflammation, reduction of vascular oxidative stress, and provides a barrier regulating vascular permeability. In the brain, the glycocalyx is closely related to functions of the blood-brain barrier and neurovascular unit, both responsible for adequate neurovascular responses to potential threats to cerebral homeostasis. An aneurysmal subarachnoid hemorrhage (aSAH) occurs following rupture of an intracranial aneurysm and leads to immediate brain damage (early brain injury). In some cases, this can result in secondary brain damage, also known as delayed cerebral ischemia (DCI). DCI is a life-threatening condition that affects up to 30% of all aSAH patients. As such, it is associated with substantial societal and healthcare-related costs. Causes of DCI are multifactorial and thought to involve neuroinflammation, oxidative stress, neuroinflammation, thrombosis, and neurovascular uncoupling. To date, prediction of DCI is limited, and preventive and effective treatment strategies of DCI are scarce. There is increasing evidence that the glycocalyx is disrupted following an aSAH, and that glycocalyx disruption could precipitate or aggravate DCI. This review explores the potential role of the glycocalyx in the pathophysiological mechanisms contributing to DCI following aSAH. Understanding the role of the glycocalyx in DCI could advance the development of improved methods to predict DCI or identify patients at risk for DCI. This knowledge may also alter the methods and timing of preventive and treatment strategies of DCI. To this end, we review the potential and limitations of methods currently used to evaluate the glycocalyx, and strategies to restore or prevent glycocalyx shedding.

## Introduction

Intracranial aneurysms (IAs) are a common vascular pathology affecting around 2.8% of the population (Vlak et al., 2011). Rupture of an IA leads to a subarachnoid hemorrhage (aSAH), which is associated with high morbidity and mortality rates (Vlak et al., 2011). Yearly, aSAH affect around 9 per 100,000 persons, with a median age of 55 years (Zacharia et al., 2010; Geraghty and Testai, 2017). Despite the advent of new therapies to treat aSAH, the clinical course of aSAH is prone to severe complications.

Initially, the rupture of an IA results in immediate damage to the brain, also referred to as early brain injury (EBI). A delayed response to EBI commonly presents between 4 and 10 days following ictus, and occurs in around 30% of aSAH (Geraghty and Testai, 2017; Peeyush Kumar et al., 2019). This response is called delayed cerebral ischemia (DCI), defined as the development of a new neurological deficit, or a decrease in the Glasgow Coma Scale by two or more points lasting for at least 1 h after exclusion of other complications (Vergouwen et al., 2010). DCI strongly affects the outcome of aSAH, as it increases morbidity and mortality rates among aSAH patients, and also prolongs hospital stays.

Until recently, DCI was considered a clinical manifestation of cerebral vasospasm, i.e., the narrowing of large intracranial arteries (Suzuki et al., 1983; Vergouwen et al., 2010). However, studies targeting vasospasm have failed to reduce the incidence of DCI. Moreover, DCI was observed in the absence of radiological vasospasms (Stein et al., 2006; Dhar and Diringer, 2015; Oka et al., 2020). These incongruences have resulted in a paradigm shift toward focusing on microvascular changes in the pathophysiology of DCI. Subsequently, several processes including neuro-inflammation, oxidative stress, neurovascular uncoupling, and microthrombosis have been related to endothelial dysfunction and the development of DCI (Budohoski et al., 2014). However, the complete pathophysiological process underlying DCI is poorly understood, thereby impeding early diagnosis and targeted treatment.

In this regard, disruption of the glycocalyx may be a relevant contributor to endothelial dysfunction and the associated processes in the development of DCI. The glycocalyx is a gel-like layer covering the luminal side of the endothelium, and is involved in numerous mechanisms regulating endothelial functions (Reitsma et al., 2007). Located between the bloodstream and the endothelium, the glycocalyx protects the endothelium and functions as an interface for integrating various signals involved in inflammation, nitric oxide (NO) release, and coagulation (Tarbell and Pahakis, 2006; Abassi et al., 2020). In recent years, the glycocalyx has gained wider attention for its pathophysiological role in the development of a multitude of vascular pathologies, like atherosclerosis and sepsis, as well as neurological disorders, like small vessel disease and epilepsy (Martens et al., 2013; Rovas et al., 2019; Yamaoka-Tojo, 2020a). Multiple studies have reported that molecules like TNF-alpha, atrial natriuretic peptide, and abnormal vascular shear stress can induce damage to the glycocalyx (Schött et al., 2016). Moreover, rodent model studies revealed that conditions like hyperglycemia, hemorrhagic shock, inflammation, and ischemia-reperfusion injuries are associated with glycocalyx shedding (Abassi et al., 2020). In addition, sepsis, trauma, and ischemia-reperfusion following aortic bypass surgery have been shown to induce glycocalyx disruption in humans (Rehm et al., 2007). The precise underlying mechanisms of glycocalyx disruption are not completely understood, but the release of a disintegrin and metalloproteinases (ADAMs), matrix metalloproteinases (MMPs) in response to inflammation or ischemia, and increased activity of heparinase and hyaluronidases have been associated with glycocalyx disruption (Ramnath et al., 2014, 2020; Goligorsky and Sun, 2020).

Since the glycocalyx is involved in mechanisms that also contribute to DCI, we hypothesize that the glycocalyx plays a role in the pathophysiology of DCI. In this review, we will discuss the role of the glycocalyx within different endothelial and microvascular processes, and relate this to the microvascular changes that have been associated with the pathophysiology of DCI. As such, we aim to evaluate the potential contribution of the glycocalyx to the development of DCI.

## Materials and Methods

Our narrative review is based on multiple search strategies addressing the various aspects of the pathogenesis of DCI following aSAH. Firstly, we sought to understand the pathophysiological mechanisms occurring at a microvascular levels following aSAH in pre-clinicals models. The search terms included “subarachnoid hemorrhage”(MESH) OR “subarachnoid hemorrhage,” OR “aneurysmal subarachnoid hemorrhage” OR “subarachnoid bleeding” combined with “experimental model” OR “animal model” OR “rodent” and “microvascular dysfunction” OR “endothelial cells” OR “microvasculature.” Papers focusing solely on vasospasms were excluded. Delayed cerebral ischemia was excluded from this search due to the difficulty of assessing neurological deterioration in animals, a prerequisite for the diagnosis of DCI. Secondly, we performed a comparable search to identify clinical studies describing pathomechanisms at the endothelial level, including the glycocalyx, and microvascular level in DCI following aSAH. The following search terms were used: “subarachnoid hemorrhage”(MESH) OR “subarachnoid hemorrhage,” OR “aneurysmal subarachnoid hemorrhage” OR “subarachnoid bleeding” combined with (“Delayed cerebral ischemia” OR “Delayed cerebral ischemia” OR “delayed ischemic neurologic deficit” OR “secondary brain ischemia” OR “secondary brain ischemia” OR “secondary cerebral deficit” OR “delayed ischemic complications”) and (“glycocalyx” OR “endothelial glycocalyx” OR “endothelial surface layer” OR “pericellular matrix” OR “endothelium” OR “endothelial cell lining” OR “microvascular dysfunction” OR “endothelial cells” OR “microvasculature” OR “microcirculation.” We applied the species filter “Humans” to this search. Based on the findings of these search strategies, we identified four main microvascular pathomechanisms underlying DCI, i.e., inflammation, oxidative stress, thrombosis, and neurovascular coupling.

Due to limited output from this search with regards to the glycocalyx, we subsequently applied search strategies focusing on the role of the glycocalyx in the delineated microvascular pathomechanisms: (“glycocalyx” OR “endothelial glycocalyx” OR “endothelial surface layer” OR “pericellular matrix”) AND (1) (“inflammation” OR “inflammatory disease”); OR (2) (“oxidative stress” OR “oxidation”); OR (3) (“coagulation” OR “coagulative state” OR “coagulative disorder” OR “thrombus” OR “thrombosis”); OR (4) (“Neurovascular unit” OR “Neurovascular coupling” OR “neurovascular decoupling”). Following title and abstract screening, the studies were included in the evidence pile per pathomechanisms, and subsequently classified as: pathophysiology-related or treatment-related. Finally, studies were included following reference screening. The search strategy was conducted by two authors (HS and RH).

## The Glycocalyx

The glycocalyx is a gel-like layer located between the endothelium and the circulating blood (Reitsma et al., 2007; Schött et al., 2016; Cosgun et al., 2020). The main constituents of the glycocalyx are proteoglycans and glycoproteins. These form the backbone of the glycocalyx, to which glycosaminoglycans are connected (Reitsma et al., 2007). The glycocalyx is a dynamic structure under constant renewal in response to hemodynamic changes, wall shear stress, and enzymatic degradation from (or by) plasma cells (Cosgun et al., 2020). Importantly, it provides a physical, cellular and electrostatic barrier that limits the passage of most plasma constituents through the endothelium (Tarbell and Pahakis, 2006). A healthy glycocalyx prevents binding of plasma cells and proteins to endothelial receptors and glycocalyx-bound molecules, thereby regulating multiple endothelial functions. As such, the glycocalyx is considered a relevant component of the blood-brain barrier (BBB) (Ando et al., 2018; Haeren et al., 2018). When in a state of disease such as in those mentioned above, the glycocalyx is disrupted, thus exposing the endothelium and its receptors to plasma cells and proteins. This leads to inflammation, reduced endothelial synthesis of nitric oxide (NO), BBB dysfunction, neurovascular uncoupling and microthrombosis.

### Glycocalyx and Inflammation

**FIGURE 1:**
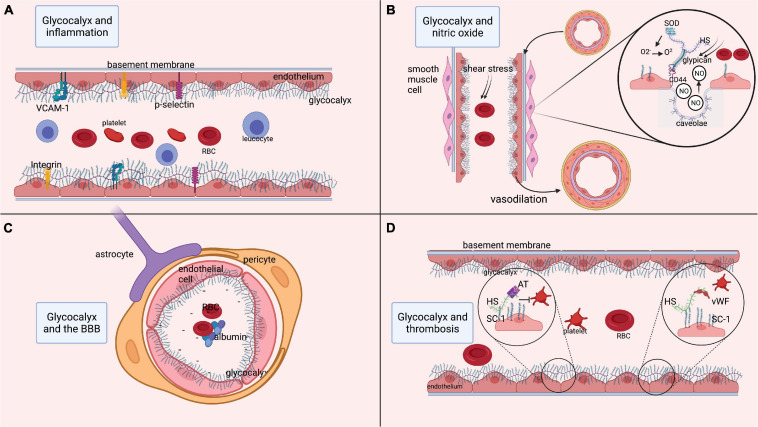
Functions of the glycocalyx. **(A)** Role of the glycocalyx in adhesion of platelets and inflammatory cells such as leukocytes. Multiple endothelial glycoproteins such as selectins, integrins, and cell-adhesion molecules (VCAM-1) are docked within the glycocalyx. As such, the glycocalyx prevents their activation and thereby prevents inflammatory processes. **(B)** Process of releasing nitric oxide (NO) from the endothelium in response to shear stress. Shear stress is sensed by heparan sulfate, which is bound to glypican. Glypican then attaches to the transmembrane CD44. This mechanical signal in response to shear stress is translated into an intracellular signaling process resulting in the release of NO from endothelial caveolae. The released NO induces relaxation of vascular smooth muscle cells, resulting in vasodilatation. Moreover, superoxide dismutase is bound to heparan sulfate and can convert oxidative O_2_^–^ into O_2_, thereby regulating the level of free radicals. **(C)** Role of the glycocalyx in maintenance of the BBB. Within cerebral microcirculation, the glycocalyx is also part of the blood-brain barrier (BBB). The BBB is further composed of tightly connected endothelial cells encapsulated by pericytes that are surrounded by astrocytic feet. At the luminal side of the endothelium, the glycocalyx layer prevents large molecules from passing the BBB. Since the glycocalyx is negatively charged, the BBB is not permeable to negatively charged cells such as platelets and red blood cells, or proteins like albumin. **(D)** Anti-coagulant function of the glycocalyx, as it docks anti-coagulant molecules like anti-thrombin, tissue factor pathway inhibitor and the von Willebrand factor (vWf). In states of increased shear stress, the vWf is elongated and able to bind platelets that in turn attach to the endothelium and initiate coagulation. AT, anti-thrombin; BBB, blood-brain barrier; ET, endothelin; GAG, glycosaminoglycan; HS, heparan sulfate; ICAM-1, inter-cellular cell-adhesion molecule 1; NO, nitric oxide; O_2_, oxygen, O_2_^–^, PG, proteoglycan; superoxide; RBC, red blood cell; VCAM-1, SC-1, syndecan-1; SOD, superoxide dismutase; vascular-cell adhesion molecule 1; vWF, von Willebrand factor. Image is created using BioRender.com.

The most important glycoproteins within the glycocalyx include selectins, integrins, and adhesion molecules (Reitsma et al., 2007; Kolářová et al., 2014). Endothelium-expressed P and E-selectins are involved in leukocyte-endothelium interaction, rolling and activation, while also promoting fibrin deposition and thrombus formation (Reitsma et al., 2007). Integrins are heterodimeric transmembrane receptors on leukocytes and platelets involved in binding these cells to the endothelium (Reitsma et al., 2007). Adhesion molecules, e.g., vascular cell adhesion molecule-1 (VCAM-1) and intercellular adhesion molecule-1 (ICAM-1), act as endothelial ligands for integrins and play a crucial role in leukocyte adhesion to the endothelium. In healthy vasculature, the glycocalyx components (thickness range: 400 – 500 nm) cover these cell-adhesion molecules (thickness range: 20 – 40 nm) on the endothelium, thereby preventing them from interacting with their ligands (Reitsma et al., 2007; Lipowsky, 2012; Choi and Lillicrap, 2020). Hence, an intact glycocalyx isolates the endothelium from circulating inflammatory cells, and limits inflammatory processes (Figure 1). When components of the glycocalyx are disrupted, the cell-adhesion glycoproteins become exposed and get activated, thus initiating a cascade of inflammatory mechanisms.

### Glycocalyx and Nitric Oxide

Nitric oxide (NO) is involved in essential functions such as regulation of smooth muscle cell relaxation, neutrophil adhesion to the endothelium, and platelet activation. In endothelial cells, NO is released from endothelial caveolae by glypican (Tarbell and Pahakis, 2006; Kumagai et al., 2009; Ebong et al., 2014; Yen et al., 2015), a membrane-bound proteoglycan that forms one of the backbones of the glycocalyx. Glypican is activated by biochemical reactions that are induced by heparan sulfate in response to changes in mechanical forces or blood flow conditions (Tarbell and Pahakis, 2006; Ebong et al., 2014; Zeng, 2017). Thus, the glycocalyx stimulates the synthesis of NO via heparan sulfate and glypican in response to an increase in shear stress, thereby regulating vasodilation (Tarbell and Pahakis, 2006). Conversely, shedding of the glycocalyx reduces shear stress-induced NO release (Yen et al., 2015). The availability and release of NO is highly relevant to ensure the continuous adjustment of local cerebral blood flow in response to the metabolic demands of neuronal tissue (Abbott et al., 2006; Stanimirovic and Friedman, 2012; Kaplan et al., 2020), a mechanism known as neurovascular coupling (NVC). As an orchestrator of NO synthesis, the glycocalyx plays a significant role in NVC, hence its degradation may lead to a reversed ischemic reaction (Kaplan et al., 2020). Moreover, the glycocalyx accommodates anti-oxidants like superoxide dismutase (SOD), which is bound to heparan sulfate (Tarbell and Pahakis, 2006). Reactive oxygen species (ROS) are usually produced in mitochondria as a product of aerobic cellular metabolism (He et al., 2017). Oxidative stress appears when ROS are insufficiently counterbalanced by antioxidants. At low levels, ROS contribute to cell processes, enabling communication between organelles and cells (Palma et al., 2020). However, at higher levels and when surpassing signaling thresholds, ROS contribute to the activation of apoptosis and autophagy, and induce damage to DNA and RNA (Palma et al., 2020). ROS include superoxide anion (O_2_^–^) and peroxynitrite (ONOO^–^) (Di Wang et al., 2010). Of these, O_2_^–^ is produced by vascular cells, for example from uncoupled endothelial nitric oxide synthase (Sena et al., 2018). This O_2_^–^ is moderately reactive by itself, but it participates in reactions with NO that result in the formation of ROS such as peroxynitritre and reactive nitrogen species, from which a diversity of other highly reactive species can be produced (Wang et al., 2018; Yin et al., 2018). Moreover, it inactivates NO and uncouples endothelial nitric oxide synthase, thereby reducing NO bioavailability (Hamed et al., 2009). Antioxidants, such as SOD, counteract and neutralize O_2_^–^ by catalyzing the dismutation of O_2_^–^ into O_2_ and H_2_O_2_, thereby preventing the formation of ONOO^–^ (Kumagai et al., 2009; Schött et al., 2016; He et al., 2017). As such, SOD represents a first-line of defense against oxygen-derived free radicals and reduces the damaging effect of superoxide (Reitsma et al., 2007; Østergaard et al., 2013; He et al., 2017). Experiments in rodents have revealed that inhibition of SOD induces endothelial dysfunction consistent with inactivation of basal NO by O_2_^–^ (Laight et al., 1998). Conversely, experimental treatment of cells with SOD attenuates O_2_^–^ production and restores NO production (Hamed et al., 2009). To conclude, the glycocalyx plays an important role in the bioavailability of endothelial NO and its functions related to NVC and oxidative stress (Figure 1).

### Glycocalyx and the Blood-Brain Barrier

The BBB is composed of a single layer of endothelial cells that are interconnected by tight junctions, surrounded by the basement membrane and pericytes, and covered by astrocytic end-feet (Sharif et al., 2018; Kaplan et al., 2020). On the luminal side of the endothelium, the glycocalyx contributes to the barrier function of the BBB (Figure 1). Using two-photon microscopy, several studies have shown that the glycocalyx acts as a barrier for large molecules (Kutuzov et al., 2018; Khan et al., 2021). The glycocalyx also functions as an electrostatic barrier to plasma proteins due to its negative charge carried by core proteins like hyaluronan, thereby repelling negatively charged cells and preventing them from binding the endothelium (Iba, 2016). Furthermore, perturbation of the glycocalyx has been shown to increase BBB permeability (Zhu et al., 2018), leading to extravasation of plasma proteins, especially albumin, which further results in astrocytic transformation and alterations in NVC (Friedman et al., 2009).

### The Glycocalyx and Coagulation

There is growing evidence that constituents of the glycocalyx also play a role in the prevention of coagulation (Figure 1). Heparan sulfate and syndecan-1 within the glycocalyx bind to anticoagulant molecules like anti-thrombin, activated protein C, and tissue factor pathway inhibitors (Reitsma et al., 2007; Iba, 2016; Peeyush Kumar et al., 2019). This leads to the production of endothelial prostacyclins that prevent platelets and neutrophils from binding to the endothelium (Iba, 2016). The glycocalyx also forms an anchorage point for the von Willebrand factor (vWF) (Choi and Lillicrap, 2020). In the presence of non-physiological or increased shear stress, the vWF changes its configuration and extends into the lumen to bind platelets. Experimental degradation of heparan sulfate from the glycocalyx has indeed revealed a decrease in vWF-induced platelet activation, underlining the role of the glycocalyx in platelet activation (Kalagara et al., 2018).

## Phases and Pathomechanisms in DCI

The period following an aSAH can be roughly divided into four different phases that are likely to contribute to the development of DCI, each characterized by various pathophysiological processes. Firstly, the initial hemodynamic changes following rupture of the IA induce *phase I: an acute inflammatory response and concurrent increased coagulation* (Sercombe et al., 2002). Thereafter, the products of inflammation in combination with the breakdown of the released erythrocytes contribute to *phase II: creating an environment high* in *oxidative stress*, which peaks at day 7 after the aSAH (Peeyush Kumar et al., 2019). Both early inflammation and oxidative stress potentiate further inflammation, ensuing *phase III: a breakdown of the* BBB coinciding with increased apoptosis of endothelial cells starting at day 7 after the ictus (Østergaard et al., 2013). Finally, all of the previous processes result in *phase IV: dysfunction of neurovascular coupling*, causing a mismatch between brain tissue metabolism and blood (oxygen) supply, which ultimately contributes to the development of DCI (Rowland et al., 2012). Based on the central role of the glycocalyx in modulating vascular inflammation, oxidative stress, BBB permeability and NVC, disintegration of the glycocalyx may play a role in the above-mentioned processes contributing to DCI. Below, we will discuss these four phases and their role in the pathophysiology of DCI in more detail.

## Phase I: Neuroinflammation and a Procoagulative State

The first phase starts directly following rupture of an IA. The rupture leads to a rapid increase in intracranial pressure, causing a decrease in cerebral blood flow and perfusion. The resulting brief cerebral circulation arrest (Terpolilli et al., 2015; Peeyush Kumar et al., 2019) causes the release of damage-associated molecular patterns (DAMPs) from necrotic cells or from extracellular matrix (Chaudhry et al., 2018; Muhammad et al., 2020). DAMPs are sensed by cerebral resident immune cells, which in turn release cytokines, like TNF-alpha, IL-1 and IL-6 (Schneider et al., 2018; Mastorakos and McGavern, 2019). These pro-inflammatory cytokines affect glycocalyx integrity, causing endothelial dysfunction and further stimulating pro-inflammatory conditions (Lipowsky, 2012; Cosgun et al., 2020). For example, cytokine-induced glycocalyx degradation exposes endothelial-bound selectins and adhesion molecules, which in turn stimulate pro-inflammatory conditions (Pradilla et al., 2010; Peeyush Kumar et al., 2019). An increased number of neutrophils and VCAM-1 in post-hemorrhagic cerebrospinal fluid (CSF) supports this idea of concomitant glycocalyx disruption and increased neuroinflammation (Nissen et al., 2001; Bell et al., 2017; Schneider et al., 2018). Moreover, clinical studies have reported increased levels of ICAM-1 in the CSF and serum of DCI patients (Nissen et al., 2001), while rodent SAH models revealed an important role of p-selectin activation in the pathophysiology of SAH-induced damage to the cerebral tissue (Ishikawa et al., 2009; Friedrich et al., 2011; Sehba and Friedrich, 2011; Atangana et al., 2017).

The increased level of p-selectin is also associated with activation and adhesion of platelets to the endothelium, and subsequently promotes fibrin deposition, platelet-leucocyte aggregation and thrombus formation (Sabri et al., 2012). Indeed, damage to the endothelium and inevitably to the glycocalyx following ictus has been associated with platelet aggregations invading the brain parenchyma in experimental rodent SAH models using rodents (Ishikawa et al., 2009; Friedrich et al., 2010; Clarke et al., 2020). Furthermore, serological measurements of pro-coagulants such as platelet activating factor (PAF), thrombin anti-thrombin complex, the vWf and fibrinogen have all been found to be increased in DCI patients compared to that of non-DCI patients (Hiroshima et al., 1997; Frijns et al., 2006; Vergouwen et al., 2008; Naraoka et al., 2014). This underlines the failure of anti-coagulants within the glycocalyx to inhibit coagulation, suggesting disruption of the glycocalyx.

Following activation of adhesion molecules, inflammatory cells get activated and release a multitude of damaging enzymes. For example, neutrophils adhere to the endothelium (Sehba et al., 2011) and release myeloperoxidase and heparinase, which are reactive oxygen species (ROS) known to disrupt the glycocalyx (Changyaleket et al., 2017; Abassi et al., 2020). This further exposes the endothelium to circulating inflammatory cells and shear stress, thereby sustaining extravasation of inflammatory cells through the endothelium (Manchanda et al., 2018). Animal models of SAH in ICAM-1 knock-out mice have revealed a decrease in the accumulation of activated microglia (Atangana et al., 2017). This finding illustrates the role of adhesion molecule activation in stimulating neuroinflammatory processes following rupture of an IA. Moreover, platelets and leukocytes recruited to the site of injury release signaling molecules such as PAF, platelet derived growth factor (PDGF) and chemokine CC motif ligand 5 (CCL5). These signaling molecules activate endothelial cells and macrophages to release additional pro-inflammatory cytokines including IL-1, IL-1β, IL-6, and TNF-α. Previous studies have shown that CSF levels of these cytokines are indeed associated with DCI in aSAH patients (Osuka et al., 1998; Sercombe et al., 2002; Budohoski et al., 2014; Bell et al., 2017). As mentioned, these cytokines degrade the glycocalyx, creating a vicious pathophysiological cycle.

## Phase II: Oxidative Stress

**FIGURE 2:**
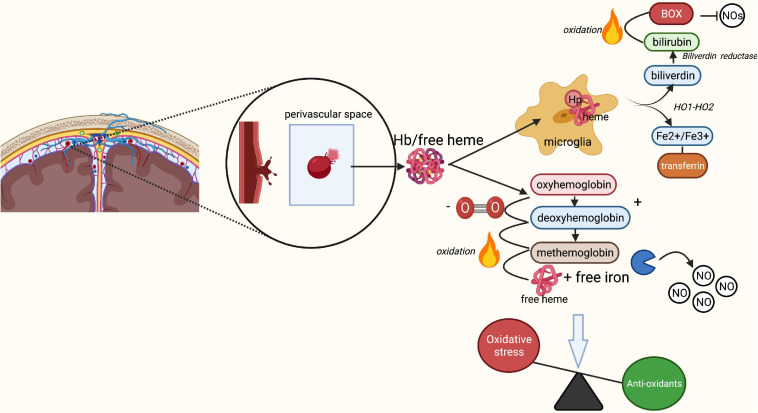
Breakdown of red blood cells following aSAH leading to oxidative stress. Rupture of an intracranial aneurysm results in the extravasation of red blood cells (RBCs) into the subarachnoid space. Here, lysis of the RBCs starts within a few days after the rupture, peaking at 7 days post-rupture. Under physiological conditions, the released free heme and iron from RBCs bind to haptoglobin, forming a Hb-Hp complex. The Hb-Hp complex subsequently attracts microglia and macrophages by the membrane receptor CD163 leading to its phagocytosis and neutralization. End products of this phagocytosis are biliverdin and iron through the action of heme oxygenase 1 and 2. Biliverdin is converted into bilirubin through biliverdin reductase. In the presence of oxygen, biliverdin may turn into bilirubin oxidation products, which act as inhibitors of nitric oxide (NO). Iron may be present in its ferrous (Fe^2+^) or ferric (Fe^3+^) form. The ferrous form of iron participates in the Fenton reaction, causing formation of reactive oxygen species and subsequent lipid peroxidation (*oxidative stress)*. Under physiological circumstances, the transport protein transferritin and binding protein ferritin prevent iron from causing damage and transport iron back to neurons for later use. In SAH, Hp quickly becomes saturated due to the enormous increase in lysis of RBCs. Unbound Hb turns into oxyhemoglobin, which loses its oxygen and converts to deoxyhemoglobin. Deoxyhemoglobin spontaneously and non-enzymatically converts to methemoglobin, which converts to free heme and iron. Free heme and iron bind NO and contribute to an absolute reduction of NO, and thus a decrease in vasodilation and increase in endothelin-induced vasoconstriction. Finally, free heme and iron contribute to the production of free radicals and lipid peroxides, increasing the overall rate of oxidative stress. BOX, bilirubin oxidation product; Fe^2+^, ferrous iron, Fe^3+^, ferric iron; Hb, hemoglobin; Hp, haptoglobin; HO-1/HO-2, heme oxygenase 1/2; NO, nitric oxide; O_2_, oxygen; ROS, reactive oxygen species. Image is created using BioRender.com.

The aSAH is associated with an increase in oxidative stress (Sehba et al., 2011), which is inversely related to the availability of NO. This increase in oxidative stress is mostly the result of the lysis of red blood cells (RBCs) released into the subarachnoid space (Sehba et al., 2011). Under physiological conditions, the extracellular RBC-derived hemoglobin (Hb) and free heme are bound to haptoglobin (Hp) that neutralizes the strong oxidative capacity of heme-iron and prepares it for phagocytosis (Rifkind et al., 2015). The Hb-Hp complex subsequently binds to the scavenger membrane receptor CD163 on macrophages and microglia, leading to its internalization (Fabriek et al., 2005; Blackburn et al., 2018). The heme is then degraded by hemeoxygenase into biliverdin and iron (Meguro et al., 2007). The latter is bound to ferritin and transported by transferritin, thereby preventing it from exercising its toxic effects (Wagner et al., 2003). The aim of this process is to prevent extracellular Hb from oxidizing, and free iron from inducing oxidation of surrounding tissue which causes the release of free radicals. However, following an aSAH, the available Hp is rapidly depleted by the sudden increase of RBCs in the subarachnoid space (Blackburn et al., 2018). As a result, only some of the Hb and free heme originating from RBCs is neutralized by Hp as discussed above (Figure 2). The remaining non-neutralized free heme and iron in the extracellular space function as highly oxidative factors, causing the formation of ROS (Macdonald and Weir, 1991; Wagner et al., 2003; Østergaard et al., 2013; Blackburn et al., 2018). Antioxidants such as NO are increasingly consumed to balance the oxidative stress in the tissue (Macdonald and Weir, 1991; Dreier et al., 2000; Sehba et al., 2011). Indeed, reduced NO synthesis has been reported following SAH in studies measuring inhibitors of nitric oxide synthesis such as Asymmetricdimethyl-l-arginine (ADMA) in cerebrospinal fluid (Jung et al., 2012; Appel et al., 2018), and in rodent SAH models measuring nitric oxide availability (Sabri et al., 2011, 2012). The final reduced NO availability is the result of unbounded extracellular Hb scavenging NO (Østergaard et al., 2013; Geraghty and Testai, 2017; Peeyush Kumar et al., 2019). This process of RBC lysis begins 2 days after the aSAH, and peaks at around day seven (Pluta et al., 1998; Østergaard et al., 2013).

Endothelial synthesis of NO is usually regulated by the ferrous dioxygen complex. However, in the presence of oxidative stress, this complex generates superoxide (O_2_^–^) which binds to NO, thereby further reducing NO availability (Sabri et al., 2011). Moreover, aSAH induces endothelial dysfunction, which limits NO synthesis and exacerbates the effects of oxidative stress (Sehba et al., 2000; Sabri et al., 2011). Finally, aSAH patients with DCI have increased levels of bilirubin oxidation products (BOXs) compared to patients without DCI (Pyne-Geithman et al., 2013). This difference is mainly dependent on levels of oxidative products in the CSF (Pyne-Geithman et al., 2013; Rapoport, 2018; Peeyush Kumar et al., 2019). Similar to free Hb, BOXs inhibit endothelial NO synthesis (Peeyush Kumar et al., 2019).

Overall, the rupture of an IA results in an excess of oxidative products that cannot be metabolized properly by the brain (Budohoski et al., 2014). This leads to a decrease in the availability of NO, which is aggravated by reduced endothelial NO synthesis. Consequently, intracellular mechanisms and metabolism are affected, leading to increased vasoconstriction with dysfunctional NVC, inflammation and lipid peroxidation. Since the glycocalyx is involved in the synthesis of endothelial NO and has additional antioxidant properties via the actions of glycocalyx-bound SOD, a damaged glycocalyx could be a potential catalysator of oxidative stress and reduced NO availability in the pathomechanisms underlying DCI (Figure 2).

## Phase III: Blood-Brain Barrier Dysfunction

Delayed cerebral ischemia has been associated with increased BBB permeability, a phenomenon mirrored by a peak of apoptotic cells around day seven post-ictus (El Amki et al., 2018). Both the initial phase I (acute inflammatory response) and the breakdown of RBCs in phase II following rupture of an IA contribute to the degradation of the BBB (Sehba et al., 2011). Products of RBC lysis serve as DAMPs and bind to innate immune cells such as microglia, which are the resident macrophage-like cells of the central nervous system (Geraghty and Testai, 2017). Microglia release inflammatory cytokines and trigger the up-regulation of endothelial adhesion molecules on the endothelium, allowing macrophages, and neutrophils to bind to the endothelium and migrate into the subarachnoid space (Atangana et al., 2017; Peeyush Kumar et al., 2019). There, they phagocytize RBCs and their released components, a process that has been correlated with DCI (Ahn et al., 2019). The accumulation of neutrophils on the endothelium also results in increased lipid peroxidation, oxidative stress and the release of enzymes that degrade the extracellular matrix, which further promote damage to the endothelium and basement membrane (Sen et al., 2006; Sabri et al., 2009; Friedrich et al., 2011; Fumoto et al., 2019). For example, upregulation of enzymes like MMP-1 and MMP-9 that are involved in remodeling of the extracellular matrix and degradation of basal membrane have been found to be good predictors of DCI when measured in plasma and CSF of aSAH patients (Fischer et al., 2013; Triglia et al., 2016; Bell et al., 2017; de Oliveira Manoel and Loch Macdonald, 2018).

Lining the luminal side of the endothelium, the glycocalyx is the first barrier to overcome for all inflammatory cells that seek to migrate into the subarachnoid space. As discussed, the glycocalyx indeed limits the passage of molecules through the endothelium, both physically and electrostatically. Previous studies using aSAH models have revealed extravasation of albumin through the BBB, suggesting increased BBB permeability (Schöller et al., 2007). Extravasation of albumin into the brain parenchyma may result in cerebral edema and astrocytic transformation, further stimulating dysfunction of NVC mechanisms (Friedman et al., 2009). These findings suggest a synergistic action of the glycocalyx in combination with BBB failure to prevent large molecules from crossing the endothelium that contributes to edema formation and eventually to a reduced blood flow manifesting itself as DCI (Tarbell and Pahakis, 2006; Østergaard et al., 2013; Alphonsus and Rodseth, 2014; Schoknecht et al., 2015; Sharif et al., 2018; Nian et al., 2020).

## Phase IV: Neurovascular Uncoupling

**FIGURE 3:**
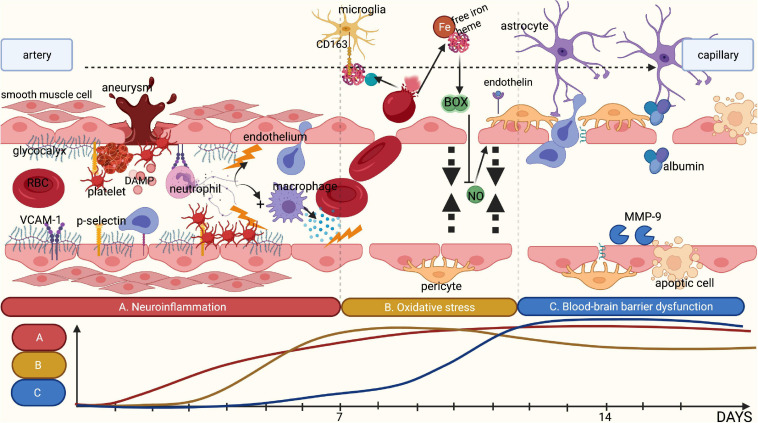
Summary of the different pathophysiological phases contributing to DCI. **(A)** Rupture of an intracranial aneurysm is associated with tissue damage and subsequent tissue ischemia, releasing DAMPs. This release attracts leucocytes such as neutrophils, thereby initiating local neuroinflammation. Combined with an increase in shear stress, rupture of an intracranial aneurysm is likely to damage the glycocalyx. This loss of glycocalyx integrity promotes adhesion of platelets and neutrophils to the endothelium by cell-adhesion molecules and selectins, which are no longer covered by the glycocalyx. The adhered inflammatory cells release cytokines, further stimulating local neuroinflammation. **(B)** Following rupture of an intracranial aneurysm, red blood cells (RBCs) enter the subarachnoid space wherein they undergo lysis, resulting in the release of free iron and heme. Under physiological conditions, haptoglobin and hemoglobin form a complex that binds microglia and macrophages through the membrane receptor CD163 for phagocytosis. However, following the rupture of an aneurysm, the copious amounts of RBC lysis products quickly saturate haptoglobin, leaving free iron unbound. Following oxidation, free iron results in the synthesis of reactive oxygen species and subsequently in reduction of the availability of nitric oxide. Moreover, the disrupted glycocalyx has a reduced synthesis capacity for nitric oxide in response to shear stress. This leads to an absolute shortage in nitric oxide, limiting vasodilatory capacity. In addition, endothelin-1 is no longer inhibited by endothelial nitric oxide leading to increased vasoconstriction. **(C)** The above-mentioned processes promote blood-brain barrier (BBB) disruption. Products of RBC lysis activate microglia, which in turn release additional cytokines. Activated neutrophils and macrophages entering the subarachnoid space also release inflammatory cytokines and matrix metalloproteinases, disrupting the extracellular matrix and the basement membrane, leading to increased BBB permeability. This is further aggravated by the disruption of the glycocalyx that can no longer exert its barrier functions. Finally, this results in the formation of local oedema, inflammation and ischemia. Image is created using BioRender.com.

The physiological process of NVC by inducing vasodilation or vasoconstriction is essential to ensure adequate blood flow to the brain parenchyma in response to changes in tissue metabolic demands. The process of NVC is regulated by the complex interaction of endothelial cells, smooth muscle cells (SMCs), neurons, microglia, pericytes and astrocytes; together these form the neurovascular unit (NVU) (Iadecola, 2004). Endothelial dysfunction and limited endothelial NO synthesis in aSAH result in an altered activation of SMCs via endothelium-released endothelin-1 and NO (Vergouwen et al., 2008; Figure 3). Moreover, astrocytic transformation due to increased BBB permeability affects intra- and extracellular water and electrolyte distributions, which in turn also affect the release of vasoactive substances such as potassium and prostacyclins (Iadecola, 2004; Koide et al., 2013b; van Dijk et al., 2016). In addition, experimentally induced inflammatory responses using lipopolysaccharide in rodents have been associated with changes in hemodynamic function and cerebrovascular dynamic, underlining the role of inflammation in dysfunction of the NVU (Brezzo et al., 2020).

The above-mentioned processes cumulatively result in dysfunction of NVC following aSAH. Preclinical and clinical studies have indeed revealed dysregulation of vasodilatation and even pathological vasoconstriction in response to different stimuli in experimental aSAH models and patients (Britz et al., 2007; Budohoski et al., 2012; Koide et al., 2013a; Balbi et al., 2017; Neumaier et al., 2021). Moreover, DCI has been associated with decreased vasoreactivity of the middle cerebral artery measured 4 to 10 days after ictus in a prospective clinical study including 34 patients with aSAH (Carrera et al., 2010). These findings are also in line with the observed increased vasoconstriction of small cortical vessels in SAH patients in response to increased levels of carbon dioxide during surgery (Pennings et al., 2004).

As a result of dysfunctional NVC, cortical spreading depolarization (CSD) may occur. This CSD is a phenomenon characterized by sudden electrical discharge due to a breakdown of ion gradients, followed by sustained neuronal and glial depolarization propagating throughout the cortex (Budohoski et al., 2012; Woitzik et al., 2012; Koide et al., 2013b). During CSD, a considerable amount of intracellular [K^+^] efflux causes an increase in perivascular (extracellular) [K^+^], which is compensated by a Ca^2+^ and Na^+^ influx into the cells (Koide et al., 2013b). As a consequence of such electrolyte shift, an uncontrolled release of excitatory neurotransmitters is triggered. Neurotransmitters depolarize neighboring neurons and cause further propagation of the depolarization wave. This spreading depolarization is associated with an arrest of electrical activity due to complete membrane depolarization, thereby inhibiting action potentials. This phenomenon has been shown to persist in the presence of ischemia and leads to a sustained vasoconstriction of cerebral vessels (Dreier et al., 2006; Bosche et al., 2010). The presence of CSD has been shown to be a good predictor of DCI, and can occur in the absence of vasospasms (Petzold et al., 2003; Dreier et al., 2006; Woitzik et al., 2012). Triggers of CSD in aSAH are thought to involve increases in extracellular (K^+^), decreased NO bioavailability, oxyhemoglobin, and endothelin-1 (Koide et al., 2013b; Østergaard et al., 2013; Geraghty and Testai, 2017). As an important regulator of NO bioavailability, and indirectly of endothelin-1, a damaged glycocalyx decreases the brain’s reserve defense mechanisms and exposes aSAH patients to processes that increase the metabolic mismatch leading to DCI. This phenomenon is further amplified by the above-mentioned mechanisms that are also likely to involve disruption of the glycocalyx: neuroinflammation, procoagulation, increase in oxidative stress and increase in BBB permeability.

## Clinical Findings on the Glycocalyx and Delayed Cerebral Ischemia

The foregoing discussion on the pathomechanisms underlying DCI illustrates that disintegration of the glycocalyx may contribute to all consecutive pathophysiological processes (Figure 3). To the best of our knowledge, there is currently only one clinical study that reported glycocalyx assessment in association with DCI (Bell et al., 2017). In this small observational study, plasma markers of glycocalyx disruption such as Syndecan-1 and CD44, a glycoprotein receptor for hyaluronan, as well as the levels of ICAM-1 and VCAM-1 were assessed in three patients who developed DCI following their aSAH. When these were compared to a healthy population, it was found that the occurrence of aSAH was associated with increased plasma levels of Syndecan-1 and CD44, as well as ICAM-1 and VCAM-1 (Bell et al., 2017). In addition, levels of Syndecan-1 increased significantly when DCI was observed (Bell et al., 2017). Since measurements of these markers were not compared to aSAH patients without DCI, it is unclear whether the increase in these markers reflects the natural course of endothelial damage following aSAH, or if it is specifically associated with endothelial damage in DCI.

Aside from this single study directly relating disruption of the glycocalyx to the development of DCI following aSAH, other studies have identified many risk factors for the development of DCI that are associated with glycocalyx disruption and dysfunction. In their review, de Rooij et al. found that, among others factors, smoking, hyperglycemia, history of diabetes mellitus, and early systemic inflammatory response syndrome were associated with the occurrence of DCI (De Rooij et al., 2013). Numerous studies have reported the destructive effects of smoking on the glycocalyx (Johnson et al., 2010; Ikonomidis et al., 2017). Similarly, hyperglycemia and diabetes mellitus have repeatedly been associated with decreased glycocalyx integrity (Lopez-Quintero et al., 2013; Kolářová et al., 2014; Yilmaz et al., 2019). These “risk factor”-based data suggest that aSAH patients who develop DCI may have a history of chronic degeneration of glycocalyx due to their generally poor vascular health, more often compared to patients who do not develop DCI. In other words, poor baseline-condition of the vasoprotective glycocalyx is a potential risk factor for aSAH-related DCI. Moreover, these vascular risk factors could explain the differences between aSAH patients. Lastly, various inflammatory conditions, including infectious diseases like Covid-19 (Yamaoka-Tojo, 2020b) and dengue fever (Puerta-Guardo et al., 2016), as well as sepsis (Rovas et al., 2018; Iba and Levy, 2019; Goligorsky and Sun, 2020) have been related to a disturbed glycocalyx integrity.

## Discussion

This narrative review sheds light on the important contributions of the glycocalyx to various physiological properties of the endothelium. Destruction of the glycocalyx and associated implications stemming from its function as a barrier and a mediator of vascular homeostasis have been demonstrated extensively. However, the clinical relevance of the glycocalyx in pathological conditions remains to be elucidated. This also applies to the potential role of the glycocalyx in the pathophysiology of DCI. In this review, we have sought to connect the physiological roles of the glycocalyx to pathomechanisms leading to DCI. However, preclinical and clinical studies relating glycocalyx perturbation and restoration to the development of DCI are sparse. This is partly due to the lack of sensitivity and specificity of the available techniques to assess glycocalyx properties (Haeren et al., 2018; Valerio et al., 2019). Therefore, this discussion will firstly focus on the strengths and limitations of the techniques available for assessing the glycocalyx of the cerebral vasculature, and secondly explore the opportunities for prevention and restoration of glycocalyx shedding.

## Techniques for Assessing the Cerebrovascular Glycocalyx

**FIGURE 4:**
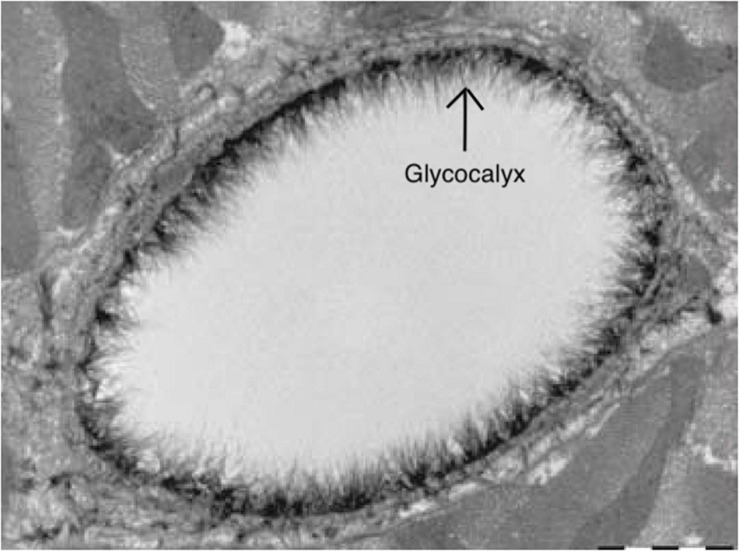
Electron microscopy image of the endothelial glycocalyx from Reitsma et al. (2007), with permission. Endothelial glycocalyx (*arrow)* of a rat left ventricular myocardial capillary following staining with Alcian blue 8GX. Image is created using BioRender.com.

Techniques for assessing the glycocalyx in *ex vivo* conditions should be discerned from those that are *in vivo*. Various microscopy techniques are available for the former techniques for assessing the cerebrovascular glycocalyx (Table 1). For example, the first images of the glycocalyx were made by Luft et al., using a ruthenium red stain with osmium tetroxide in combination with transmission electron microscopy (Luft, 1966). Although the glycocalyx can indeed be visualized with transmission electron microscopy (Figure 4), this technique warrants many preparation procedures that affect constituents of the glycocalyx, causing it to collapse (Haeren et al., 2016). This collapse can be partly overcome by rapid freezing and a freeze substation technique, but this is likely to underestimate the actual glycocalyx thickness, which complicates comparisons (Ebong et al., 2011). Other *ex vivo* microscopy techniques such as confocal and two-photon laser scanning microscopy are mainly used to visualize specific constituents of the glycocalyx using fluorescent markers like lycopersicon esculentum agglutinin or wheat germ agglutinin (Reitsma et al., 2007). This enables both a quantitative (i.e., glycocalyx thickness) and qualitative (i.e., constituent ratios) three-dimensional analysis of the glycocalyx (Reitsma et al., 2011). Furthermore, stochastic optical reconstruction microscopy was recently used to reveal specific glycocalyx components with very high resolution (Xia and Fu, 2018; Fan et al., 2019). However, these techniques are nevertheless limited by the frailty of the glycocalyx in *ex-vivo* conditions (Reitsma et al., 2007; Haeren et al., 2016). Recent advances in handheld multi-photon microscopy probes could potentially provide a novel *in vivo* technique to evaluate the glycocalyx (Khan et al., 2021).

**TABLE 1:** Summary of the pros and cons of different methods for assessing the glycocalyx.

	TEM	CLSM	TPLSM	SDF	OPS	Markers
	
	*Ex vivo*	*Ex vivo*	*Ex vivo*	*In vivo*	*In vivo*	*In vitro*
Advantages	Quantitative assessment	Quantitative and qualitative assessment	Quantitative and qualitative assessment	No collapse Quantitative assessment No dye	No collapse Quantitative assessment No dye	Quantitative assessment
Limitations	Time consuming Glycocalyx collapse	Use of lectins Glycocalyx collapse	Use of lectins Glycocalyx collapse	Motion and pressure artifacts	Motion and pressure artifacts	Non-specific Invasive

Current *in vivo* techniques are mainly based on handheld videomicroscopy techniques, such as orthogonal polarization spectral (OPS) imaging and its successor, sidestream darkfield (SDF) imaging. In both techniques, light is emitted at a wavelength of 530nm, which is absorbed by (de-)oxyhemoglobin within erythrocytes (Nieuwdorp et al., 2008) that are observed as small discs flowing through the microcirculation. Based on the width variations of the intraluminal red blood cell column, the perfused boundary region – a validated marker for glycocalyx thickness – is calculated (Goedhart et al., 2007; Nieuwdorp et al., 2008; Lee et al., 2014). These techniques have been applied in cerebral microcirculation during intracranial surgery, including aneurysm surgery (Pennings et al., 2004; Pérez-Bárcena et al., 2011; Haeren et al., 2018). However, cerebral microcirculation is not accessible for follow-up measurements after surgery, limiting its clinical application. Assessment of the sublingual microcirculation is non-invasive and easily accessible, and therefore often used as a surrogate measurement for systemic glycocalyx evaluation (Nieuwdorp et al., 2008). This has indeed been applied successfully, and revealed that various vascular disease-related risk factors like hypertension and atherosclerosis, as well as specific diseases like renal failure, sepsis, infections and lacunar stroke, are associated with decreased glycocalyx thickness (Vlahu et al., 2012; Dane et al., 2013; Martens et al., 2013; Becker et al., 2015; DellaValle et al., 2019; Yamaoka-Tojo, 2020a; Weinbaum et al., 2021). With regards to assessment of cerebral microcirculation, recent studies have applied SDF imaging techniques to the conjunctiva of the eye, and related this to cerebral pathology (Tamosuitis et al., 2016). As conjunctival microcirculation is supplied by branches of the ophthalmic artery, this microcirculation may resemble the pathology of cerebral microcirculation seen in DCI. As such, evaluation of the glycocalyx of conjunctival microcirculation could provide a non-invasive surrogate measure of the glycocalyx of cerebral microcirculation.

Finally, another technique to evaluate glycocalyx integrity is the measurement of structural and specific constituents of the glycocalyx, such as Syndecan-1, Heparan sulfate and Hyaluronan, within blood plasma or CSF. Numerous studies have shown that these glycocalyx markers are increased in the presence of inflammation, sepsis, hypoperfusion, and ischemic stroke (Rehm et al., 2007; Ostrowski et al., 2015; DellaValle et al., 2019; Abassi et al., 2020). The major advantage of these analyses is their simplicity and safety. However, these analyses are limited by high inter-individual variability and lack of specificity (Haeren et al., 2016).

## Prevention and Restoration of Glycocalyx Shedding

If glycocalyx shedding precedes the occurrence of DCI, preventing this shedding could be a novel strategy for arresting the development of DCI. Similarly, considering disintegration of the glycocalyx as a pathomechanism of DCI, restoration of the glycocalyx could provide a potential therapeutic strategy for DCI. Multiple studies have explored the potential of prevention and restoration of glycocalyx shedding in laboratory and preclinical conditions. Among these, sphingosine-1 phosphate (S1P) is one of the most studied potential molecules. S1P is a sphingolipid in the blood plasma that is usually incorporated within serum albumin and high-density lipoproteins (Zeng et al., 2014). It inhibits the actions of MMPs, which results in a reduction of Syndecan-1 shedding (Curry et al., 2012; Zeng et al., 2014). Moreover, S1P induces glycocalyx synthesis on cultured endothelial cells (Zeng et al., 2015); in combination with heparan sulfate, S1P has the potential to regenerate the glycocalyx (Mensah et al., 2017). S1P mediates its actions via S1P receptors on the endothelial surface (Lee et al., 1998). In previous studies, S1P administration indeed preserved the glycocalyx in endothelial cell monolayers and in rats’ microvessels, while concurrently maintaining normal vascular permeability (Zhang et al., 2016; Mensah et al., 2017). Furthermore, the infusion of 5% albumin – a major source of S1P – as fluid resuscitation following hemorrhagic shock restored glycocalyx thickness, reduced plasma syndecan-1 to baseline levels, and stabilized microvascular permeability and leucocyte adhesion in rats (Torres et al., 2017; Aldecoa et al., 2020). Since fluid therapy is central in the treatment of SAH and prevention of DCI (Tseng et al., 2008; Diringer et al., 2011), a comprehensive understanding of the effects of different kinds of fluid therapies on the glycocalyx could offer additional benefits.

Direct inhibitors of MMPs such as biphenylylsulfonylamino-3-phenylpropionic acid and batimastat have also been shown to be effective in restoring glycocalyx integrity after its breakdown (Ramnath et al., 2014, 2020). Furthermore, hydrocortisone and dexamethasone have succesfully been applied to reduce glycocalyx degradation, possibly by reducing local inflammation and indirectly inhibiting MMPs (Chappell et al., 2007; Cui et al., 2015; Zhu et al., 2018; Yu et al., 2019). For example, Zhu et al. described the protective effects of dexamethasone on the integrity of the cerebral endothelial glycocalyx in mice, thereby also reducing BBB leakage and the occurrence of cerebral edema (Zhu et al., 2018).

In addition to MMP inhibition, numerous pharmacological strategies have been applied to prevent glycocalyx shedding or restore glycocalyx integrity, with some promising – albeit preliminary – results. These include anti-diabetic medication like sulodexide, atrasentan and metformin (Boels et al., 2016; Haare et al., 2017), anticoagulant medication like antithrombin (Becker et al., 2010), and the stimulation of angiogenesis by growth factors (Desideri et al., 2019).

Although the above-mentioned results on strategies for preventing and restoring glycocalyx disruption seem promising, we did not find any clinical studies that evaluated their effects on the glycocalyx, or on disease conditions. Therefore, these findings should be considered as preliminary, and should be supported by further studies.

## Future Perspectives

Based on our proposed model relating the glycocalyx to DCI, it remains to be determined whether glycocalyx shedding precedes or results from the pathophysiological processes of DCI. Shedding could be a result of the aforementioned pathophysiological mechanisms of DCI, making evaluation of glycocalyx integrity a potential biomarker of DCI. Alternatively, glycocalyx shedding could be a primary contributor to the development of DCI, which would imply that prevention and restoration of glycocalyx shedding are a potential novel preventive and therapeutic targets for DCI, respectively. Evaluation of the glycocalyx, or glycocalyx markers in preclinical models of aSAH could form a future direction for DCI studies. However, preclinical studies are lacking a standardized approach to mimic aSAH, and mainly evaluate acute or short-term changes following the induction of the subarachnoid hemorrhage, thus limiting their ability to evaluate changes related to DCI. Moreover, preclinical models may not be comparable to the pathophysiology of DCI in humans. In the absence of a standardized preclinical model mimicking human aSAH and the development of DCI, clinical studies may offer more potential to evaluate the glycocalyx in relation to the development of DCI. To date, only one small (*n* = 3) clinical study has observed the relation between the glycocalyx and occurrence of DCI following aSAH (Bell et al., 2017). Hence, further and larger clinical studies evaluating glycocalyx integrity in aSAH patients are needed to determine a potential relation, and the preventive and/or therapeutic value of the glycocalyx as a biomarker for the development of DCI. Currently, one registered ongoing clinical study (NCT03706768) is analyzing glycocalyx changes in an aSAH cohort, however, the results have not been published yet. Considering the lack of sensitivity and specificity of the current glycocalyx analysis methods, we would recommend applying multiple glycocalyx assessment techniques simultaneously. Hence, a large observational study in which glycocalyx markers are frequently evaluated in serum and/or CSF of aSAH patients, of which around 30% will develop DCI, seems a reasonable approach to explore a role of the glycocalyx in the development of DCI. In addition, the assessment of the microcirculation and its glycocalyx using SDF-imaging could form an additional strategy in these patients. In this regard, one could consider intraoperative cerebral measurements, and pre- and postoperative sequential imaging of the sublingual and conjunctival microcirculation. Findings from such clinical studies could steer the direction of future studies. Such future studies could for example assess the potential of preventive measures of glycocalyx disruption and the therapeutic value of glycocalyx restoration in patients suffering from DCI. Importantly, further studies are needed to evaluate the actual therapeutic properties of the pharmacological substances regenerating or protecting the glycocalyx, as current evidence is limited.

### Limitations

This study has multiple limitations. Firstly, this review is not a systematic review, and the search strategy was based on multiple explorative search queries, thereby limiting reproducibility and introducing a selection bias. The explorative nature of this study, also resulted in a process of study selection that is subject to an inherent selection bias. Furthermore, only one small case study described original experiments relating glycocalyx markers to the development of DCI, whereas other studies included in this review mainly provided indirect evidence to support our hypothesis. This reduces the strength of our conclusion. Moreover, the complexity of this topic, i.e., pathomechanisms of DCI following aSAH, is further increased by the lack of standardized and reliable preclinical study models to mimic the effects of aSAH in humans. Consequently, the preclinical studies included in our review used multiple aSAH models in numerous animals, mainly rodents, which may not be comparable to the clinical situation. Lastly, definitions of DCI have evolved over time (from vasospasms to delayed ischemic neurological deficit), preventing accurate charting and comparative analysis of results of studies evaluating the pathophysiology of DCI after aSAH.

## Conclusion

We have reviewed the physiological roles of the glycocalyx with the aim of relating these to the pathomechanisms involved in DCI. Our results suggest that glycocalyx damage is associated with the development of DCI, however, current studies on this association are sparse. To explore this hypothesis, clinical studies assessing glycocalyx properties of the cerebral microcirculation in aSAH patients are warranted. Since the techniques that are currently available for evaluating the glycocalyx are lacking sensitivity and specificity, the inclusion of multiple assessment techniques would be indispensable for such studies.

## Author Contributions

HS is the first author of this work. HS and RH wrote the manuscript. RH has senior authorship of this work. EN, OT, ID, JD, MN, YT, and GH have critically reviewed the manuscript. All authors contributed to the article and approved the submitted version.

## Conflict of Interest

The authors declare that the research was conducted in the absence of any commercial or financial relationships that could be construed as a potential conflict of interest.

## Publisher’s Note

All claims expressed in this article are solely those of the authors and do not necessarily represent those of their affiliated organizations, or those of the publisher, the editors and the reviewers. Any product that may be evaluated in this article, or claim that may be made by its manufacturer, is not guaranteed or endorsed by the publisher.
